# Family, personal, parental correlates and behavior disturbances in school-aged boys with attention-deficit/hyperactivity disorder (ADHD): a cross-sectional study

**DOI:** 10.1186/s13034-022-00467-w

**Published:** 2022-04-19

**Authors:** Yuan-Chang Hsu, Chih-Tsai Chen, Hao-Jan Yang, Pesus Chou

**Affiliations:** 1 Department of Child and Adolescent Psychiatry, Tao-Yuan Psychiatric Center, Ministry of Health and Welfare, 71 Long-Shou St. Tao-Yuan District, Taoyuan City, Taiwan; 2 Institute of Public Health, National Yang Ming Chiao Tung University, Hsinchu, Taiwan; 3 Department of Public Health, Chung-Shan Medical University, Taichung, Taiwan; 4 Community Medicine Research Center, National Yang Ming Chiao Tung University, Hsinchu, Taiwan

**Keywords:** Attention-deficit/hyperactivity disorder (ADHD), Problem behavior, School children, Male

## Abstract

**Background:**

To evaluate the relationship among family, personal, parental correlates, and behavioral disturbances in boys with attention-deficit/hyperactivity disorder (ADHD).

**Methods:**

We performed a hospital-based cross-sectional study. School-aged boys who first visited the hospital between 2000 and 2011 with ADHD were identified. Through medical records review, demographic information, family characteristics, personal characteristics, parental characteristics, and the Child Behavior Checklist (CBCL) collected during the first outpatient visit were retrieved. A T-score higher than 63 in the internalizing or externalizing subscale of the CBCL indicated severe behavioral disturbances in each domain. Multivariable logistic regression was used to evaluate the relationship between the correlates and behavioral disturbances. Eligible patients were further classified into groups without behavioral disturbance, with either only severe internalizing or only severe externalizing behaviors, or with both behaviors. Multivariable ordinal logistic regression was used to investigate the association between the correlates and the number of types of behavioral disturbances.

**Results:**

A total of 1855 boys with ADHD were included. In the multivariable logistic regression, family factors, including being first-born, living in a family not with both parents, and family history of mental disorder, were associated with severe internalizing and externalizing behaviors. Personal factors, including prenatal complications, perinatal complications, and medical and psychiatric comorbidities, were associated with severe internalizing behaviors, but only prenatal complications and medical comorbidities were associated with severe externalizing behaviors. Parental factors were only associated with severe externalizing behaviors. A higher paternal education level had a protective effect, but younger motherhood increased the risk. In ordinal logistic regression, these factors were also associated with more types of behavioral disturbances.

**Conclusions:**

Multiple factors are related to behavioral disturbances in ADHD. Our study reported the association among family, personal, parental factors, severe internalizing behavior, severe externalizing behavior, and number of behavioral disturbances in boys with ADHD. However, the impacts differed as the behavior phenotypes varied. Further research is needed to better understand the heterogeneity of ADHD behavior.

## Introduction

Attention-deficit/hyperactivity disorder (ADHD), a common psychiatric disorder among children, is associated with long-term negative consequences [1–4]. Coexisting emotional and behavioral disturbances may exaggerate the negative influence of ADHD on children, resulting in further impairment. Conceptually, children’s emotional and behavioral disturbances can be categorized into internalizing and externalizing behaviors. Internalizing behaviors are linked to over-controlled and inner-directed problems [5, 6], manifesting as symptoms of anxiety, depression, or withdrawal behavior [5–7]. Externalizing behaviors often involve inadequate regulation, such as behavior disinhibition or outward behaviors [5, 7, 8]. The presentation of internalizing or externalizing behaviors contributes to the heterogeneous behavioral phenotypes of children. Children with ADHD are more vulnerable to the occurrence and influence of problematic internalizing and externalizing behaviors. Children with ADHD with severe internalizing or externalizing behaviors often experience more negative outcomes than those without such behaviors [9]. The risk of other risk-taking behaviors also increases [9–11]. Moreover, behavioral disturbances in ADHD may result in work incapacity, inappropriate sexual behaviors, excessive medical utilization, or other detrimental life outcomes and persist into adulthood [12–15]. Because of the negative impact of internalizing and externalizing behaviors on child development, the evaluation of behavioral disturbances in children with ADHD is important.

The pathogenic mechanism of behavioral disturbances in children with ADHD is complex. Although most contemporary research on ADHD focuses on biological factors such as genetic characteristics and structural or functional abnormalities of the brain, the role of socioenvironmental factors should not be underrated. Although several different classes of genome variants have been found to be associated with ADHD, some observational epidemiological studies have also shown that exposure to environmental toxins, dietary factors, low income, and family adversity is related to ADHD [16]. Other studies also reported that numerous demographic, personal, and parental factors all influence child development [17–19]. Previous studies have reported the impact of prenatal and perinatal complications, such as maternal physical, social, or psychological problems during pregnancy, complicated delivery, low birth body weight, and jaundice on child behaviors [17]. The effect of family structure and birth order on child aggressive behaviors was also reported in a hospital-based study [20]. The influence of parental characteristics on child development has also been emphasized in many studies [21–23]. However, the effect of socioenvironmental factors on the heterogenous behavioral phenotypes of ADHD is scarcely investigated. Furthermore, although most studies have been performed in Western countries, few have been conducted in Asian populations.

## Methods

### Aim

The aim of this study was to evaluate the relationship among family, personal, parental factors, and behavioral disturbances among school-aged boys with ADHD using a medical record database from a public psychiatry hospital in Taiwan, a country in the Asia-Pacific region nurtured by Eastern culture.

### Data source

The Tao-Yuan Psychiatric Center (TYPC) is a public psychiatric hospital located in northern Taiwan. Covered by the National Health Insurance system, TYPC provides services for patients with mental disorders with a catchment area for more than 2 million residents. The Department of Child and Adolescent Psychiatry in TYPC provides psychiatric service for patient younger than 18 years. A comprehensive assessment is performed on the first visit. First, a questionnaire about demographic, personal, parental, and family information of the child and the Child Behavior Checklist (CBCL) must be completed by the caretaker. Second, the main caretaker is interviewed by a trained interviewer to verify the collected information. Third, a semi-structured interview based on the diagnostic criteria of the *Diagnostic and Statistical Manual of Mental Disorders, 4th Edition, Text Revision* (DSM-IV-TR), is performed by board-certified psychiatrists with both the child and the main caretaker to make a diagnosis and decide further treatment plans. All data collected are stored in either electronic or paper forms in the medical record database.

### Study design and participants

We performed a hospital-based cross-sectional study using the TYPC medical records database. First, we identified outpatients who first visited the Child and Adolescent Psychiatry Department between 2000 and 2011. Second, male patients aged 6 to 11 years at the first visit were considered. Third, patients receiving either a primary or secondary diagnosis of ADHD (ICD-9-CM: 314.XX) at the first visit were included in the study. No exclusion criteria were applied. After ascertainment of our study population, the medical records of eligible patients were reviewed. Information including personal characteristics, parental characteristics, family characteristics, diagnosis at the first visit, and the CBCL were collected for analysis.

## Measurements

### Family factors

Family factors, including birth order (first-born or not), sibling status (with or without siblings), family structure (with both parents or not with both parents), and family history of mental disorders (with or without a family history of mental disorders), were collected and dichotomously classified. The characteristics of the family structure, such as living in a family with both parents, with single parents, with only grandparents or other relatives, or living in a foster institute, were also collected and classified into categories as living in a family with both parents and not with both parents.

### Personal factors

Demographic information regarding age and sex was collected. Additional information during prenatal and perinatal periods, including birth mode (categories as normal spontaneous delivery or cesarean section), birth term (categorized as full-term if the child was delivered after a pregnancy for more than 37 weeks, or alternatively as preterm), birth body weight (categorized as normal if the birth weight was equal or more than 2500 g or alternatively as low birth weight), prenatal complications (categorized as having any prenatal complication or not), and perinatal complications (categorized as having any perinatal complications or not) were also collected. Medical or psychiatric comorbidities recorded during the first visit were also collected and categorized as having comorbid diseases.

### Parental factors

Information about parental characteristics, including education level, occupational status, and age at childbirth, were also collected. Parental educational level was classified as receiving education for more than 12 years. Parental occupational status at the first visit was classified as being employed or not. Parental age at childbirth was classified as younger than 20 years of childbirth or not. Both paternal and maternal information were collected.

### Behavioral disturbances

The CBCL, which includes items on a three-point Likert scale, is a common instrument for measuring problematic behaviors in children. In this study, the CBCL was used to assess behavioral disturbances in boys with ADHD [24, 25]. First, the CBCL completed by the caretaker during the first visit was retrieved from the medical record database. Second, the raw scores of the internalizing and externalizing subscales were calculated and transformed into T scores according to norms established in a previous domestic study in Taiwan. Anxious/depressed, withdrawn/depressed, and somatic complaint subscales were included in the internalizing subscale. The rule-breaking and aggressive behavior subscales were included in the externalizing subscale. A T score higher than 63 in the internalizing or externalizing subscale indicated the existence of severe behavioral disturbances in each domain. Boys with ADHD were then categorized as having severe internalizing behaviors or not and having severe externalizing behaviors or not for further analysis. Eligible patients were further categorized according to the number of types of severe behavioral disturbances. Patients with a T score lower than 63 in both internalizing and externalizing subscales were classified as the group without severe behavioral disturbance. Patients with T scores higher than 63 in only one of the internalizing or externalizing subscales were classified as having one type of behavioral disturbance, that is, either only severe internalizing behaviors or only severe externalizing behaviors. Patients with T scores higher than 63 in both internalizing and externalizing subscales were classified as having two types of behavioral disturbances, that is, both severe internalizing and externalizing behaviors. This classification indicated the presence of diversities of behavioral disturbances in boys with ADHD.

### Statistical analysis

Descriptive statistics were computed, and the differences between school-aged boys with ADHD with and without internalizing or externalizing behaviors were compared. Categorical variables were analyzed with chi-square tests, whereas continuous variables were analyzed using Student’s *t*-test.

The associations between correlates and behavioral disturbances among school-aged boys with ADHD were assessed using logistic regression. Both unadjusted and adjusted estimates with simultaneous control of all relevant variables were reported. Ordinal logistic regression was performed to assess the association between the correlates and numbers of types of behavioral disturbances. Both crude and adjusted estimates were reported.

A two-tailed *P* value < 0.05 was considered statistically significant. Association estimates were indexed as odds ratios (ORs) and 95% confidence intervals (CIs). All statistical analyses were performed using SAS version 9.4 (SAS Institute, Inc., Cary, NC, USA).

## Results

A total of 1855 school-aged boys with ADHD were included in our study. The mean age was 8.3 years. A total of 818 boys (44.1%) had severe internalizing behaviors, 982 boys (52.9%) had severe externalizing behaviors, 552 (29.8%) had only one type of behavioral disturbance in either severe internalizing or externalizing behaviors, and 624 (33.6%) had behavioral disturbances in both classes. The characteristics of the participants are presented in Table 1. Compared with school-aged boys without severe internalizing behaviors, those with severe internalizing behaviors were more likely to be first-born, to be living in a family not with both parents, to have a family history of mental disorders, to have prenatal complications, to have perinatal complications, to have medical comorbidities, and to have psychiatric comorbidities. There were no significant differences in the parental characteristics.

**Table 1:** Characteristics of school-aged boys with ADHD with and without severe internalizing/ externalizing behaviors

Variables	Internalizing behaviors (n = 1855)					Externalizing behaviors (n = 1855)				
	Severe (n = 818)		Not severe (n = 1037)		*P* value	Severe (n = 982)		Not severe (n = 873)		*P* value
	n	(%)	n	(%)		n	(%)	n	(%)	
***Family factors***										
**Birth order**					< 0.001					0.02
First-born	502	61.4	548	52.8		580	59.1	470	53.8	
Later-born	316	38.6	489	47.2		402	40.9	403	46.2	
**Family structure**					0.006					< 0.001
Not with both parents	197	24.1	188	18.1		247	25.1	144	16.5	
With both parents	621	75.9	849	81.9		735	74.9	729	83.5	
**Sibling status**					0.22					0.12
Without siblings	157	19.2	176	17.0		189	19.3	144	16.5	
With siblings	661	80.8	861	83.0		793	80.7	729	83.5	
**Family history of mental disorders**					0.002					0.001
Yes	402	49.1	435	41.9		478	48.7	359	41.1	
No	416	50.9	603	58.1		504	51.3	514	58.9	
***Personal factors***										
**Birth term**					0.36					0.08
Preterm	102	12.5	115	11.1		127	12.9	90	10.3	
Full-term	716	87.5	922	88.9		855	87.1	783	89.7	
**Birth mode**					0.35					0.20
Normal spontaneous delivery	308	37.7	369	35.6		345	35.1	332	38.0	
Cesarean section	510	62.3	668	64.4		637	64.9	541	62.0	
**Birth body weight (BBW)**					0.46					0.21
< 2500 g	55	6.7	61	5.9		68	6.9	48	5.5	
≥ 2500 g	763	93.3	976	94.1		914	93.1	825	94.5	
**Prenatal complication**					< 0.001					0.03
Yes	171	20.9	132	12.7		178	18.1	125	14.3	
No	647	79.1	905	87.3		804	81.9	748	85.7	
**Perinatal complication**					< 0.001					0.06
Yes	266	32.5	248	23.9		290	29.5	224	25.7	
No	552	67.5	789	76.1		692	70.5	649	74.3	
**Medical comorbidity**					< 0.001					< 0.001
Yes	177	21.6	147	14.2		203	20.7	121	13.9	
No	641	78.4	890	85.8		779	79.3	752	86.1	
**Psychiatric comorbidity**					< 0.001					0.65
Yes	414	50.6	425	41.0		449	45.7	390	44.7	
No	404	49.4	612	59.0		533	54.3	483	55.3	
***Parental factors***										
**Paternal education level**					0.98					< 0.001
> 12 years	338	41.3	429	41.4		355	36.1	412	47.2	
≦12 years	480	58.7	608	58.6		627	63.9	461	52.8	
**Maternal education level**					0.64					< 0.001
> 12 years	302	36.9	372	35.9		317	32.3	357	40.9	
≦12 years	516	63.1	665	64.1		665	67.7	516	59.1	
**Paternal occupational status**					0.10					0.15
Employed	791	96.7	987	95.2		935	95.2	843	96.6	
Unemployed	27	3.3	50	4.8		47	4.8	30	3.4	
**Maternal occupational status**					0.83					0.20
Employed	530	64.8	677	65.3		652	66.4	555	63.6	
Unemployed	288	35.2	360	34.7		330	33.6	318	36.4	
**Paternal age at childbirth**					0.11					0.34
< 20 years	8	1.0	4	0.4		8	0.8	4	0.5	
≥ 20 years	810	99.0	1033	99.6		974	99.2	869	99.5	
**Maternal age at childbirth**					0.08					0.002
< 20 years	38	4.7	32	3.1		50	5.1	20	2.3	
≥ 20 years	780	95.3	1005	96.9		932	94.9	853	97.7	

With regard to externalizing behaviors, school-aged boys with ADHD with such behaviors were more likely to be first-born, to be living in a family not with both parents, to have a family history of mental disorders, to have prenatal complications, and to have medical comorbidities than those without such behaviors. Moreover, boys with ADHD with severe externalizing behavior problems were more likely to have parents with a lower education level and to be born to mothers younger than 20 years.

Table 2 presents the estimate of logistic regression among family, personal, and parental factors and severe internalizing behaviors in school-aged boys with ADHD. Being first-born, living in a family not with both parents, having family history of mental disorders, having prenatal complications, having perinatal complications, having medical comorbidities, and having psychiatric comorbidities were associated with severe internalizing behaviors in boys with ADHD. The role of the parental factors was not significant.

**Table 2:** Family factors, personal factors, parental factors, and severe internalizing behaviors among school-aged boys with ADHD

Variables	Crude estimate	Adjusted estimate
	OR [95% CI]	OR [95% CI]
***Family factors***		
**Birth order**		
First- vs. later-born	1.41 [1.18, 1.71]***	1.45 [1.17, 1.79]***
**Family structure**		
Not with vs. with both-parents	1.43 [1.11, 1.87]**	1.30 [1.03, 1.46]*
**Sibling status**		
Without vs. with siblings	1.16 [0.92, 1.47]	0.94 [0.73, 1.23]
**Family history of mental disorders**		
Yes vs. no	1.34 [1.11, 1.61]**	1.26 [1.04, 1.53]*
***Personal factors***		
**Birth term**		
Preterm vs. full-term birth	1.14 [0.86, 1.52]	0.93 [0.61, 1.42]
**Birth mode**		
Cesarean section vs. normal spontaneous delivery	1.09 [0.90, 1.32]	1.05 [0.86, 1.28]
**Birth body weight**		
< 2500 vs. ≥2500 g	1.15 [0.79, 1.68]	0.93 [0.61, 1.42]
**Prenatal complication**		
Yes vs. no	1.81 [1.41, 2.32]***	1.72 [1.33, 2.22]***
**Perinatal complication**		
Yes vs. no	1.53 [1.25, 1.88]***	1.42 [1.14, 1.78]**
**Medical comorbidity**		
Yes vs. no	1.67 [1.31, 2.13]***	1.51 [1.18, 1.94]***
**Psychiatric comorbidity**		
Yes vs. no	1.48 [1.23,1.78]***	1.49 [1.23, 1.81]***
***Parental factors***		
**Paternal education level**		
> 12 vs. ≤ 12 years	0.99 [0.83, 1.20]	0.96 [0.76, 1.20]
**Maternal education level**		
> 12 vs. ≤ 12years	1.05 [0.87, 1.27]	1.07 [0.84, 1.35]
**Paternal occupational status**		
Yes vs. no	1.48 [0.92, 2.39]	1.59 [0.97, 2.61]
**Maternal occupational status**		
Yes vs. no	0.98 [0.81, 1.19]	0.99 [0.81, 1.21]
**Paternal age at childbirth**		
< 20 vs. ≥20 years	2.55 [0.77, 8.50]	1.62 [0.44, 6.03]
**Maternal age at childbirth**		
< 20 vs. ≥20 years	1.53 [0.95, 2.47]	1.30 [0.76, 2.24]

* *P* < 0.05

** *P* < 0.01

*** *P* < 0.001

In the analysis of severe externalizing behaviors, most results were similar to internalizing behaviors. However, some differences, particularly the effects of parental factors, existed (Table 3). Being first-born, living in family not with both parents, having a family history of mental disorders, prenatal complications), and having medical comorbidities were associated with severe externalizing behavior problems in boys with ADHD. However, the association among perinatal complications, psychiatric comorbidities, and severe externalizing behaviors was not significant. Moreover, a higher paternal education level was associated with a lower risk and maternal age younger than 20 years at childbirth was associated with a higher risk of severe externalizing behaviors only. This association was not noted in internalizing behaviors.

**Table 3:** Family factors, personal factors, parental factors and severe externalizing behaviors among school-aged boys with ADHD

Variables	Crude estimate	Adjusted estimate
	OR [95% CI]	OR [95% CI]
***Family factors***		
**Birth order**		
First-born vs. Later-born	1.24 [1.03, 1.49]*	1.28 [1.04, 1.58]*
**Family structure**		
Not with vs. with both-parents	1.70 [1.34, 2.14]***	1.35 [1.04, 1.74]*
**Sibling status**		
Without vs. with siblings	1.21 [0.95, 1.53]	0.97 [0.74, 1.27]
**Family history of mental disorders**		
Yes vs. no	1.36 [1.13, 1.63]***	1.31 [1.09, 1.58]**
***Personal factors***		
**Birth term**		
Preterm vs. full-term birth	1.29 [0.97, 1.72]	1.22 [0.88, 1.68]
**Birth mode**		
Cesarean section vs. normal spontaneous delivery Deliv	0.88 [0.73, 1.06]	0.84 [0.69, 1.03]
**Birth body weight**		
< 2500 vs. ≥2500 gm	1.28 [0.87, 1.87]	1.10 [0.72, 1.67]
**Prenatal complication**		
Yes vs. no	1.33 [1.03, 1.70]*	1.33 [1.03, 1.73]*
**Perinatal complication**		
Yes vs. no	1.21 [0.99, 1.49]	1.10 [0.88, 1.38]
**Medical comorbidity**		
Yes vs. no	1.62 [1.27, 2.07]***	1.49 [1.15, 1.91]**
**Psychiatric comorbidity**		
Yes vs. no	1.04 [0.87, 1.25]	1.01 [0.84, 1.22]
***Parental factors***		
**Paternal education level**		
> 12 vs. ≦12 years	0.63 [0.53, 0.76]***	0.72 [0.58, 0.91]**
**Maternal education level**		
> 12 vs. ≦12years	0.68 [0.57, 0.83]	0.83 [0.66, 1.05]
**Paternal occupational status**		
Yes vs. no	0.71 [0.44, 1.13]	0.81 [0.50, 1.31]
**Maternal occupational status**		
Yes vs. no	1.13 [0.94, 1.37]	1.16 [0.95, 1.42]
**Paternal age at childbirth**		
< 20 vs. ≥20 years	1.78 [0.54, 5.95]	1.71 [0.19, 2.71]
**Maternal age at childbirth**		
< 20 vs. ≥20 years	2.29 [1.35, 3.87]**	1.78 [1.08, 3.21]*

* *P* < 0.05

** *P* < 0.01

*** *P* < 0.001

Table 4 reports the results of ordinal logistic regression that assessed the association between the correlates and the numbers of types of behavioral disturbances in boys with ADHD. Family factors including being first-born, living in family not with both parents, and family history of mental disorders were associated with more types of behavioral disturbances. The role of personal factors, including prenatal complications, perinatal complications, psychiatric comorbidities, and medical comorbidities were also identified. With parental factors, higher paternal education level was related to a lower risk of more types of behavioral problems in boys with ADHD. Maternal age younger than 20 years at childbirth was associated with a higher risk. The role of the other parental factors was not significant.

**Table 4:** Ordinal logistic regression of risk factors and numbers of types of behavioral disturbances among school-aged boys with ADHD

Variables	Crude estimate	Adjusted estimate
	OR [95% CI]	OR [95% CI]
***Family factors***		
**Birth order**		
First vs. later-born	1.35 [1.14, 1.60]***	1.40 [1.16, 1.69]***
**Family structure**		
Not with vs. with both-parents	1.38 [1.12, 1.71]**	1.17 [1.12, 1.54]*
**Sibling status**		
Without vs. with siblings	1.20 [0.97 1.50]	0.95 [0.74, 1.21]
**Family history of mental disorders**		
Yes vs. no	1.38 [1.17, 1.64]***	1.31 [1.10 1.57]**
***Personal factors***		
**Birth term**		
Preterm vs. full-term birth	1.24 [0.95, 1.60]	1.06 [0.79, 1.42]
**Birth mode**		
Cesarean section vs. normal spontaneous delivery	0.98 [0.82, 1.17]	0.93 [0.78, 1.11]
**Birth body weight**		
< 2500 vs. BBW ≥ 2500 g	1.24 [0.88, 1.75]	1.01 [0.69, 1.48]
**Prenatal complication**		
Yes vs. no	1.62 [1.29, 2.03]***	1.61 [1.06, 2.12]***
**Perinatal complication**		
Yes vs. no	1.40 [1.16, 1.69]*	1.29 [1.06, 1.58]**
**Medical comorbidity**		
Yes vs. no	1.72 [1.38, 2.15]***	1.57 [1.25, 1.97]***
**Psychiatric comorbidity**		
Yes vs. no	1.26 [1.07. 1.50]**	1.24 [1.05, 1.48]*
***Parental factors***		
**Paternal education level**		
> 12 vs. ≦12 years	0.77 [0.66, 0.92]**	0.81 [0.66, 0.95]*
**Maternal education level**		
> 12 vs. ≦12years	0.83 [0.70, 0.99]	0.93 [0.75, 1.15]
**Paternal occupational status**		
Yes vs. no	1.02 [0.67, 1.55]	1.14 [0.74, 1.76]
**Maternal occupational status**		
Yes vs. no	1.06 [0.89 1.26]	1.07 [0.90, 1.29]
**Paternal age at childbirth**		
< 20 vs. ≥20 years	2.19 [0.74, 6.47]	1.06 [0.33, 3.44]
**Maternal age at childbirth**		
< 20 vs. ≥20 years	1.95 [1.24, 3.04]*	1.56 [1.15, 2.57]*

* *P* < 0.05

** *P* < 0.01

*** *P* < 0.001

## Discussion

This study evaluated the relationship among family, personal, parental factors, and behavioral disturbances among school-aged boys with ADHD using medical records from a public psychiatry hospital in Taiwan, and the results showed that family, personal, and parental factors contribute to the occurrence and heterogeneity of behavioral disturbances in school-aged boys with ADHD. Both internalizing and externalizing behaviors are associated with family factors, including being first-born, living in a family not with both parents, and family history of mental disorders. Among personal factors, prenatal complications, perinatal complications, and medical or psychiatric comorbidities are associated with severe internalizing behaviors, but only prenatal complications and medical comorbidities are associated with severe externalizing behaviors. Regarding parental factors, higher paternal education level is protective, whereas maternal age younger than 20 at childbirth increases the risk of externalizing behaviors; however, such an association is not noted with regard to severe internalizing behaviors. Assessment of the association between the correlates and the numbers of types of behavioral disturbances showed that the results were similar.

In our study, several family factors were associated with severe internalizing behaviors, externalizing behaviors, and the number of types of behavioral disturbances. The negative effect of an altered family structure on child development has been reported in previous studies [26, 27]. Living in a family not with both parents is associated with limited social resources, lower family support levels, and poorer family functioning [28]. The altered family structure also causes higher stress for caretakers [29, 30]. Problems of adjustment and self-regulation have also been reported to be common among children raised in families without an intact structure [29, 31]. All these disadvantages can establish a negative environment for children with ADHD and lead to severe behavioral disturbances through the interaction of the environment and impulsive tendency, which is consistent with our findings.

Family history of mental disorders and being first-born are two other important family factors associated with severe behavioral disturbances in boys with ADHD. Several hypothetical mechanisms can explain how a family history of mental disorders influences child behaviors. First, the effect of genetic transmission of mental disorders may have a direct influence on child behaviors, resulting in both the incidence and coexisting behavioral disturbances in ADHD [32–35]. Second, psychopathology in family members may have an adverse effect on parenting skills, the family atmosphere, parent–child relationship, adaptive strategies to child behaviors, and medical resource utilization [36], which are all important for children with ADHD. Third, family cohesion is disturbed by other mentally ill members. The child may imitate behaviors through modeling or social learning processes [37, 38]. Such behaviors may be used to elicit attention from others or to compensate for the feeling of being neglected. Consequently, a series of inappropriate behaviors may occur [37].

Being first-born is another risk factor for the development of severe behavioral disturbances in boys with ADHD. Several studies have reported that the first-borns often face unique challenges, such as the changed interaction between parents after the birth of a new child or the issue of sibling rivalry as they grow up [39]. In addition, inexperienced child rearing, attitude, and the capability of the parents also influence the behavior development of the first-born child [40, 41]. Additionally, first-born boys are often viewed as the heirs of the family in Eastern countries. Coexisting ADHD in first-born boys may result in experiences of failure, frustration, and greater stress. Internalizing and externalizing behaviors can, therefore, be a way for them to cope with such difficulties.

Prenatal and perinatal complications can interfere with the normal development of children through changes in both neurophysiological mechanisms and the dopaminergic system [42, 43]. Our study also reports that prenatal and perinatal complications are associated with behavioral disturbances in boys with ADHD, which is consistent with other studies [44]. However, although prenatal complications are associated with both internalizing and externalizing behaviors, perinatal complications are associated with internalizing behaviors only. Previous studies have also shown that prenatal and perinatal complications occurring during different stages of child development may have different effects on child behavior [45, 46]. This helps explain the discrepancies observed in our study.

Comorbid conditions are also related to behavioral disturbances in boys with ADHD. This result can be explained in several ways. First, from the perspective of children, having coexisting diseases may cause distress [47–49]. The interaction between distress and impulsive tendencies related to ADHD may result in further behavioral disturbances. Second, from the perspective of caretakers, a higher rearing burden may be related to a sense of fatigue and exhaustion [50, 51], interfering with parent–child interaction and child development. In our study, medical comorbidities were associated with both internalizing and externalizing behaviors in boys with ADHD, but psychiatric comorbidities were associated with internalizing behaviors only. Although this finding might be affected by a type II error, the discrepancy also implies that the relationship between comorbid status and ADHD outcomes may be more complex and requires further evaluation.

Two parental factors, paternal education level and maternal age at childbirth, are associated with the occurrence of severe externalizing behaviors and heterogeneity in behavioral disturbances in boys with ADHD. The higher education level of parents is associated with efficient parenting, flexibility in interactions, stress buffering, and management of children’s problems [52–55]. Therefore, it is reasonable that a higher parental education level is protective against severe externalizing or more problematic behaviors in children with ADHD. ADHD is often related to academic achievement. Parents who do not attain higher education levels may also have ADHD-like traits or similar behavioral problems. The possible transmission of susceptibility genes to their children may also help to explain the relationship [56].

The association between parental age at childbirth and behavioral disturbances can be explained by common genetic and socioeconomic mechanisms [57]. Parents giving birth to younger children may have heritable impulsive or novelty-seeking characteristics and influence their offspring [57, 58]. Disadvantaged families, lower social support, financial or social difficulties, and disturbed maturation of parents are also associated with early childbearing. Such disadvantages may interfere with the quality of nourishing and maturity of young parents [56, 58], resulting in both the incidence of ADHD and consequential behavioral disturbances in their children [58, 59].

Although our findings support the association between parental education level and age of childbirth on behavioral disturbances in boys with ADHD, the results between parents vary. The effect of parental gender on child development has been discussed in several studies. Parental gender may play a role in social development, quality of life, and family interactions of the child [21, 22, 60]. One study also emphasized the effects of parental education level on the general well-being of children with specific diseases [61]. While explaining our results, some cultural-specific characteristics of Asian families should be considered. In Eastern culture, paternal and maternal roles in social participation, social expectation, labor diversion in child-rearing, and decisions regarding family affairs differ. Fathers are often dominant and the authority figure in Eastern families, explaining the significant role of paternal education level in our findings. Furthermore, teenage pregnancy and early motherhood often face higher social criticism, less support, and more stress in Eastern society, constituting an unfavorable environment for child nourishment and development. Such a phenomenon can explain the influence of early motherhood on child behavioral disturbances. Although the impact of cultural context can be used to explain our results, further investigations are needed.

Our study has some limitations. First, because of the cross-sectional study design, the causal relationship should be interpreted conservatively. Second, the study population was identified from a hospital-based medical record database, and only male patients were included. The representativeness of our study was limited. In addition, the study population was recruited from a public psychiatric hospital. In Taiwan, disadvantaged families, which are also a risk factor for more severe disturbances in children, often visit public hospitals for medical treatment. This may result in referral bias in our study and interfere the inference of our results. Third, some possible unmeasured confounders, such as child temperament, parenting patterns, or family atmosphere, were not considered. Therefore, residual confounding effects still exist.

Regardless of these limitations, our study has several strengths. First, our study provides a broader perspective on ADHD, focusing on the severity and diversity of behavioral disturbances. Second, our study highlights the important role of environmental factors in children with ADHD. Third, our results reflect the association between the correlates and child behaviors in Eastern society, which has been less investigated in previous studies. Fourth, our findings can be used to identify higher-risk school-aged boys with ADHD who may have more negative outcomes and need further intervention.

## Conclusions

ADHD is a highly heterogeneous condition, and the unique family, personal, and parental characteristics of children with ADHD who have behavioral disturbances should be identified. Our study reports that family structure, birth order, family history of mental disorders, prenatal complications, perinatal complications, comorbid status, paternal education level, and maternal age at childbirth are associated with severe and heterogeneous behavioral disturbances in boys with ADHD. More research is needed to elucidate the underlying mechanism for early identification of children with ADHD at risk, adequate treatment referral, and resource allocation.

## Data Availability

The datasets analyzed during the current study are not publicly available because of the regulations of the institutional review board, but are available from the corresponding author upon reasonable request.

## Abbreviations

ADHD: Attention-deficit/hyperactivity disorder
CBCL: Child Behavior Checklist
CI: 95% confidence interval
DSM-IV-TR: *Diagnostic and Statistical Manual of Mental Disorders, 4th Edition, Text Revision*
OR: Odds ratio
TYPC: Tao-Yuan Psychiatric Center
